# Role of placental glucose metabolism in steroidogenesis in healthy pregnancies at term

**DOI:** 10.1007/s00404-025-08293-z

**Published:** 2026-04-28

**Authors:** Muhammad Umar Sajjad, Tore Henriksen, Guttorm Haugen, Trond M. Michelsen

**Affiliations:** 1 Department of Obstetrics, Division of Obstetrics and Gynecology, Oslo University Hospital, Rikshospitalet, Sognsvannsveien 20, 0372 Oslo, Norway; 2 Institute of Clinical Medicine, University of Oslo, Oslo, Norway; 3 Department of Fetal Medicine, Division of Obstetrics and Gynecology, Oslo University Hospital, Rikshospitalet, Oslo, Norway

**Keywords:** Steroidogenesis, Placenta, Metabolites, Steroids, Glucose

## Abstract

**Background:**

The human placenta consumes, on average, one third of the glucose from maternal blood. However, the role of placental glucose consumption in the production of estradiol and progesterone remains unclear. We hypothesized that placental glucose consumption in humans is associated with steroid production via a non-glycolytic pathway.

**Methods:**

We included 41 healthy pregnancies at term. Blood samples were obtained from the maternal radial artery, uterine vein, and from the umbilical artery and vein during scheduled cesarean delivery. Blood flow in the uterine artery and umbilical vein was measured using Doppler ultrasound. Plasma concentrations of estradiol, progesterone, glucose, insulin, lactate, and ketones were analyzed. We calculated uteroplacental uptake and consumption of maternal glucose and ketones, and the loss of uteroplacental lactate in 6-carbon units as well as the release of steroid hormones into maternal circulation.

**Results:**

Our data revealed a net placental release of estradiol and progesterone into maternal circulation [24.1 (5.34, 49.8) and 560.3 (61.2, 798.2) nmol/min, respectively]. The release of estradiol was positively associated with uteroplacental glucose uptake (ρ = 0.59, *p* < 0.001) and consumption (ρ = 0.43, *p* = 0.005), while progesterone exhibited similar associations (ρ = 0.61, *p* < 0.001; ρ = 0.43, *p* = 0.005). Notably, both hormones correlated positively with lactate-adjusted uteroplacental glucose consumption but not with acetate-equivalent uteroplacental ketone consumption.

**Conclusion:**

Placental release of estradiol and progesterone correlates with uteroplacental consumption of glucose that primarily occurs via non-glycolytic pathways in the third trimester placenta.

## What does this study add to the clinical work?

**Table:** 

Our study establishes a close relationship between uteroplacental consumption of maternal glucose and the placental release of estradiol and progesterone into maternal circulation, with indications that steroid production is primarily linked to the pentose phosphate pathway rather than glycolysis. These findings suggest that maternal energy status may directly affect placental steroidogenesis, given that the placenta predominantly relies on maternal glucose for its energy needs.	

## Introduction

Female steroid hormones such as estrogens and progesterone play crucial roles in maintaining normal pregnancy and intrauterine environment. Alterations in their levels are associated with pregnancy complications such as preterm birth, preeclampsia, and gestational diabetes mellitus [1–5].

The human placenta becomes the main source of female steroid hormones during pregnancy after the first trimester [6, 7]. These hormones have been reported to modulate uteroplacental vasculature [8], an essential component of maternal–fetal transport. Estrogens are also known to be key regulators of energy homeostasis and metabolic health, and estrogen deficiency has been linked to obesity and metabolic disorders [9, 10]. It has also been suggested that estrogens play crucial roles in fetal growth and development as well as fetal metabolism [11]. Furthermore, reduced placental estrogen production has been linked to placental insufficiency [12].

The villous trophoblasts are the placental cells that are predominantly responsible for the metabolism of maternal–fetal substrates, including glucose, lactate, lipids, and amino acids, as well as for gas exchange and the clearance of fetal waste products [6]. The synthesis of steroids requires access to reducing compounds, like nicotinamide adenine dinucleotide phosphate (NADPH) [13]. In vivo, the human placenta exhibits high glycolytic activity with net production of lactate that consumes the reduced NADH [14–18]. The human placenta consumes considerable amounts of maternal acetate and ketones that may be oxidized in the citric acid cycle providing reduced NADH and NADPH [14, 19, 20]. There is also evidence that the pentose phosphate pathway, which provides NADPH, is active in the placenta [21]. It is, however, unclear how glycolysis, consumption of acetate, and the pentose pathway are related to steroid production in the human placenta in vivo. Based on the general insight into steroid synthesis, we hypothesized that uteroplacental glucose uptake and consumption are associated with placental steroidogenesis, but are unrelated to placental glycolysis and acetate consumption when assessed in vivo in humans.

## Methods

### Participants

This is a cross-sectional study from our 4-vessel study cohort, and we included forty-one participants with healthy pregnancies at term with complete steroid hormone data from maternal radial artery and uterine vein, Doppler ultrasound blood flow measurements of uterine artery and umbilical vein, and glucose measurements of maternal and fetal vessels [14, 22, 23]. All samples were drawn at scheduled cesarean delivery after obtaining written informed consent. The indications for cesarean delivery were maternal requests, previous cesarean section, other uterine procedures, suspected fetopelvic disproportion, and other reasons. The exclusion criteria for this study cohort have previously been reported [23, 24].

### Blood flow measurements

Blood flow in the maternal uterine artery and umbilical vein were measured as reported previously [22, 23]. Briefly, on the day of delivery, blood vessel diameters and time-averaged maximum velocity (TAMX) measurements were recorded by the same examiner (GH) using the same equipment (Acuson Sequoia 512; Siemens Healthcare GmbH, Erlangen, Germany) as described previously [22, 23]. Thereafter, blood flow volume (mL/min) was quantified by employing the following equation,

$$\text{Blood flow},\left[Q\right] \left(\mathrm{ml}/\mathrm{min}\right)= \left(h \times {\left[\frac{D}{2}\right]}^{2}\times \pi \, \times \text{ TAMX}\right),$$

where *h* is coefficient for the spatial blood velocity profile, *D* is diameter of the vessel, and TAMX is time-averaged maximum velocity.

The coefficients of 0.5 and 0.6 were utilized for umbilical vein and uterine arteries, respectively, as reported previously [22, 25].

### 4-vessel blood sampling

We obtained blood samples from both maternal and fetal vessels (maternal radial artery, maternal uterine vein, and umbilical artery and umbilical vein) between 2012 and 2016 using the 4-vessel sampling approach as reported earlier [22]. We processed blood samples from all four vessels in separate tubes for plasma, as previously reported [22]. Briefly, blood samples collected in plasma tubes were allowed to rock for 1–2 min before being centrifuged at 2500*g* for 20 min at 6 °C. Subsequently, supernatants from plasma samples were collected and stored at − 80 °C. We used plasma samples for glucose, insulin, lactate, ketones, and steroid hormone measurements.

### Lactate and ketone measurements

Lactate and ketone concentrations (acetate, acetoacetate, and β-hydroxybutyrate) were measured in both maternal and fetal plasma samples using a quantitative proton (^1^H) nuclear magnetic resonance (NMR) spectroscopy-based method at an accredited laboratory (Nightingale Health, Finland). The NMR protocol includes deproteinization and dilution in a suitable buffer for optimal measurement. After preparation, samples are placed in an NMR tube and analyzed using a high-field NMR spectrometer, enabling direct quantification of metabolites through their chemical shifts and peak area integration in the resulting spectra. A detailed protocol for this method has been published previously [14, 26, 27].

### Steroid hormones analysis

Both estradiol and progesterone levels were measured in the maternal radial artery and uterine vein using well-established electrochemiluminescence immunoassays (Elecsys Estradiol III and Progesterone III, Cobas®, respectively) in an accredited laboratory at Oslo University Hospital, Oslo, Norway [22].

### Blood glucose and insulin measurements

Maternal and fetal plasma glucose and insulin concentrations were measured by utilizing hexokinase/glucose-6-phosphate dehydrogenase enzymatic assay and the electrochemiluminescence immunoassays, respectively, in an accredited laboratory at Oslo University Hospital, Oslo as reported previously [23, 24].

### Calculations

We used the following equations to calculate the placental release of steroid hormones into the maternal circulation, uteroplacental uptake, consumption, and fetal uptake of metabolic substrates.

$$\text{Maternal venous}-\text{arterial difference} \,  \left(\mathrm{nmol}/\mathrm{L}\right) = {\left[S\right]}_{\mathrm{MV}}- {\left[S\right]}_{\mathrm{MA}},$$

$$\text{Net placental steroid hormone release} \,  \left(\mathrm{nmol}/\mathrm{min}\right)= \left(\left({\left[S\right]}_{\mathrm{MV}}- {\left[S\right]}_{\mathrm{MA}}\right)\times {Q}_{\mathrm{M}}\right)\div 1000,$$

$$\text{Net uteroplacental uptake }\left(\mu \mathrm{mol}/\mathrm{min}\right)= \left({\left[S\right]}_{\mathrm{MA}}- {\left[S\right]}_{\mathrm{MV}}\right)\times {Q}_{\mathrm{M}},$$

$$\text{Net fetal uptake} \,   \left(\mu \mathrm{mol}/\mathrm{min}\right) = \left({\left[S\right]}_{\mathrm{fv}}- {\left[S\right]}_{\mathrm{fa}}\right)\times {Q}_{\mathrm{f}} ,$$

$$\text{Net uteroplacental consumption }\left(\mu \mathrm{mol}/\mathrm{min}\right)= \left(\left({\left[S\right]}_{\mathrm{MA}}- {\left[S\right]}_{\mathrm{MV}}\right)\times {Q}_{\mathrm{M}}\right)- \left(\left({\left[S\right]}_{\mathrm{fv}}- {\left[S\right]}_{\mathrm{fa}}\right)\times {Q}_{\mathrm{f}}\right),$$

where [*S*] represents the concentrations of a measured metabolic substrate (glucose, lactate, and ketones) or steroid hormone.

$$\text{Lactate adjusted uteroplacental glucose consumption } \left(\mu \mathrm{mol}/\mathrm{min}\right)= \left({\left[\mathrm{Glu}\right]}_{\mathrm{MA}}- {\left[\mathrm{Glu}\right]}_{\mathrm{MV}}\right)\times {Q}_{\mathrm{M}}-\left({\left[\mathrm{Glu}\right]}_{\mathrm{fv}}- {\left[\mathrm{Glu}\right]}_{\mathrm{fa}}\right)\times {Q}_{\mathrm{f}}+ \left(\left({\left[\mathrm{La}\right]}_{\mathrm{MA}}- {\left[\mathrm{La}\right]}_{\mathrm{MV}}\right)\times {Q}_{\mathrm{M}}-\left({\left[\mathrm{La}\right]}_{\mathrm{fv}}- {\left[\mathrm{La}\right]}_{\mathrm{fa}}\right)\times {Q}_{\mathrm{f}}\right)\div 2,$$

where [Glu] and [La] represent glucose and lactate concentrations, respectively. MA and MV represent the maternal radial artery and uterine vein, respectively. fv and fa represent the umbilical vein and umbilical artery, respectively. *Q*_M_ is the blood flow of the maternal uterine artery and *Q*_f_ represents the blood flow of the umbilical vein. In this equation, the placental glucose consumption is adjusted for net lactate production [14].

Acetate-equivalent uteroplacental ketone consumption was calculated by converting acetoacetate and 3-hydroxybutyrate into carbon-atom equivalents to acetate followed by combining uteroplacental consumption of acetate and acetate-equivalent ketones as reported previously [14].

### Statistics

In this study, normally distributed variables were expressed as mean and standard deviations (SD). Medians with first (Q1) and third (Q3) quartiles were used for all non-normally distributed variables as well as for those with outliers. We provided numbers and percentages for categorical variables. We employed Spearman’s rank correlation analyses to investigate the relationships between different variable pairs in this study, and the *p* value of ≤ 0.05 was considered a significant correlation. The Statistical Package for the Social Sciences (SPSS) version 29 was used for all statistical analyses, and scatter plots were produced using Graphpad Prism (version 10.1.2) software. We have also utilized the BioRender website (www.biorender.com) to create the diagram for this study.

## Results

The average age of the participants in the study was 35.8 years, the median gestational age was 39.3 weeks, and 27% of the participants were nulliparous. The median pre-pregnancy body mass index was 21.9 kg/m^2^ (range 20.3, 23.5) and the mean gestational weight gain was 14.5 kg (± 4.43), respectively. Maternal and fetal characteristics, along with measurements of glucose, insulin, and blood flow, are presented in Table 1.

**Table 1:** Maternal and fetal characteristics data, steroid hormones analyses, and metabolic substrates measurements

	Mean (SD)/Median (Q1, Q3)/[*n* = 41]	No. (%)
*Maternal characteristics*		
Maternal age (years)	35.8 (3.66)	
Nulliparous (%)		11 (26.8)
Higher education (%)		35 (85.4)
Pre-pregnancy non-smoking (%)		35 (85.4)
Employment (%)		37 (90.2)
Pre-pregnancy BMI (kg/m^2^) [*n* = 37]	21.9 (20.3, 23.5)	
Gestational weight gain (GWG) [*n* = 37]	14.5 (4.43)	
Maternal glucose (mmol/L), radial artery	4.46 (0.36)	
Maternal insulin (pmol/L), radial artery	55.4 (27.0)	
Maternal glucose (mmol/L), uterine vein	4.16 (0.33)	
Maternal insulin (pmol/L), uterine vein	40.6 (20.3)	
*Fetal and neonatal clinical characteristics*		
Gestational age (weeks)	39.3 (38.9, 39.4)	
Birthweight (g)	3440.6 (466.6)	
Placental weight (g)	545.4 (110.7)	
Sex, males (%)		26 (63.4)
Fetal heart rate (BPM)	135.0 (129.0, 142.5)	
Fetal glucose (mmol/L), umbilical artery	3.15 (0.31)	
Fetal insulin (pmol/L), umbilical artery [*n* = 39]	59.5 (36.2, 92.2)	
Fetal glucose (mmol/L), umbilical vein	3.77 (3.57,4.00)	
Fetal insulin (pmol/L), umbilical vein	56.1 (37.4, 91.4)	
*Estradiol, progesterone, and metabolic substrates measurements*		
Estradiol (nmol/L), maternal radial artery	76.2 (57.8, 92.0)	
Estradiol (nmol/L), maternal uterine vein	130.6 (94.0, 183.8)	
Estradiol venous–arterial difference (nmol/L)	57.5 (13.4, 110.0)	
Net placental release of estradiol (nmol/min)	24.1 (5.34, 49.8)	
Progesterone (nmol/L), maternal radial artery	713.3 (492.6, 975.9)	
Progesterone (nmol/L), maternal uterine vein	1740.5 (887.8, 2417.2)	
Progesterone venous–arterial difference (nmol/L)	1152.2 (160.8, 1520.1)	
Net placental release of progesterone (nmol/min)	560.3 (61.2, 798.2)	
Uteroplacental glucose uptake (µmol/min)	131.3 (62.0, 216.7)	
Uteroplacental glucose consumption (µmol/min)	17.1 (− 47.5, 78.1)	
Fetal glucose uptake (µmol/min)	126.7 (75.8, 170.2)	
Acetate-equivalent uteroplacental ketone consumption (µmol/min) [*n* = 31]	36.7 (3.37, 91.5)	
Lactate-adjusted uteroplacental glucose consumption (µmol/min) [*n* = 31]	15.6 (− 67.9, 71.7)	

**Fig. 1:**
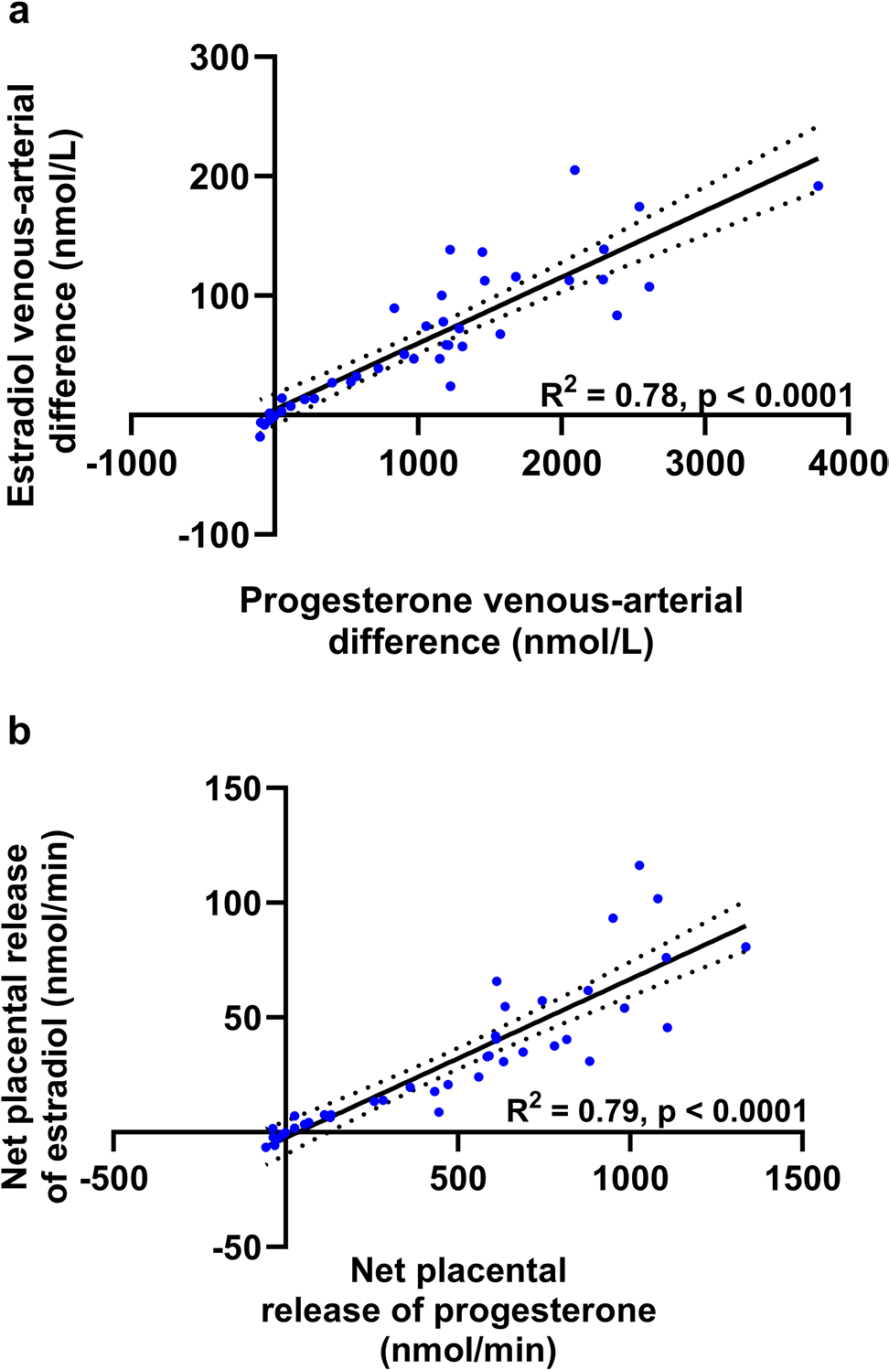
Relationship between the placental release of estradiol and progesterone into the maternal circulation. **A** A positive association between venous–arterial difference of estradiol and venous–arterial difference (maternal uterine vein–radial artery) of progesterone (nmol/L) (*n* = 41). **B** A positive association between the net placental release of estradiol and the net placental release of progesterone (nmol/min) into the maternal circulation (*n* = 41). Scatter plots were produced using Graphpad Prism software

Steroid hormones including estradiol and progesterone are mainly produced by the placenta during pregnancy and released into maternal circulation. The median estradiol and progesterone venous-arterial (VA) differences were 57.5 (13.4, 110.0) nmol/L and 1152.2 (160.8, 1520.1) nmol/L, respectively. The net placental release of estradiol and progesterone was 24.1 (5.34, 49.8) nmol/min and 560.3 (61.2, 798.2) nmol/min, respectively. The VA difference of estradiol was highly correlated with the VA difference of progesterone (nmol/L) (ρ = 0.91, *p* < 0.001, *n* = 41). Similarly, the net placental release of estradiol showed a positive correlation with the net placental release of progesterone into the maternal circulation (ρ = 0.95, *p* < 0.001, *n* = 41, Fig. 1). The net placental release of estradiol and progesterone into the maternal circulation was not significantly correlated with maternal age, parity, pre-pregnancy BMI, gestational weight gain, gestational age, or placental and birthweight measures. Notably, the net release of placental estradiol or progesterone into the maternal circulation was inversely correlated with glucose levels in the uterine vein (*p* < 0.05). In contrast, no such correlation was observed in the maternal radial artery (Table 2).

**Table 2:** Correlations of net placental release of steroid hormones with maternal and fetal parameters

	Net placental release of estradiol (nmol/min)			Net placental release of progesterone (nmol/min)		
	ρ	*p* value	*n*	ρ	*p* value	*n*
Age (years)	0.24	0.13	41	0.19	0.23	41
Parity	0.20	0.21	41	0.16	0.31	41
Pre-pregnancy BMI (kg/m^2^)	− 0.06	0.73	37	− 0.09	0.59	37
Gestational weight gain (kg)	− 0.11	0.53	37	− 0.001	0.997	37
Gestational age (weeks)	− 0.28	0.08	41	− 0.23	0.15	41
Placental weight (g)	0.01	0.95	41	0.12	0.45	41
Birthweight (g)	0.11	0.51	41	0.18	0.25	41
Maternal glucose (mmol/L), radial artery	− 0.17	0.28	41	− 0.13	0.42	41
Maternal glucose (mmol/L), uterine vein	− 0.41	0.008	41	− 0.38	0.01	41
Maternal insulin (pmol/L), radial artery	− 0.08	0.64	41	− 0.07	0.65	41
Maternal insulin (pmol/L), uterine vein	− 0.05	0.77	41	− 0.07	0.66	41

In vivo, the human placenta exhibits high glycolytic activity with net production of lactate as well as consuming considerable amounts of maternal ketones [14, 16–18]. We, therefore, explored to which extent these pathways were related to maternal steroid hormone levels and placental release of steroid hormones (Table 3). Estradiol levels in the uterine vein were positively correlated with uteroplacental glucose uptake (ρ = 0.45, *p* = 0.003, *n* = 41) and consumption (ρ = 0.32, *p* = 0.04, *n* = 41), but not with fetal glucose uptake (ρ = 0.04, *p* = 0.79, *n* = 41). In addition, the net release of estradiol into the maternal circulation was also positively correlated with uteroplacental glucose uptake (ρ = 0.59, *p* < 0.001, *n* = 41) and consumption (ρ = 0.43, *p* = 0.005, *n* = 41) but not with fetal glucose uptake (ρ = 0.05, *p* = 0.78, *n* = 41). Similarly, progesterone levels in the uterine vein were positively correlated with uteroplacental glucose uptake (ρ = 0.52, *p* < 0.001, *n* = 41) and consumption (ρ = 0.40, *p* = 0.01, *n* = 41). Furthermore, the net placental release of progesterone into the maternal circulation was positively correlated with uteroplacental glucose uptake (ρ = 0.61, *p* < 0.001, *n* = 41) and consumption (ρ = 0.43, *p* = 0.005, *n* = 41) and not with fetal glucose uptake (ρ = 0.08, *p* = 0.60, *n* = 41).

**Table 3:** Correlation of steroid hormones with uteroplacental glucose uptake, consumption, and fetal uptake (*n* = 41)

	Uteroplacental glucose uptake (µmol/min)		Uteroplacental glucose consumption (µmol/min)		Fetal glucose uptake (µmol/min)	
	ρ	*p* value	ρ	*p* value	ρ	*p* value
Estradiol (nmol/L), maternal radial artery	0.11	0.50	0.05	0.74	0.10	0.54
Estradiol (nmol/L), maternal uterine vein	0.45	0.003	0.32	0.04	0.04	0.79
Estradiol venous–arterial difference (nmol/L)	0.50	< 0.001	0.37	0.02	-0.03	0.87
Net placental release of estradiol (nmol/min)	0.59	< 0.001	0.43	0.005	0.05	0.78
Progesterone (nmol/L), maternal radial artery	0.09	0.57	0.11	0.51	-0.02	0.92
Progesterone (nmol/L), maternal uterine vein	0.52	< 0.001	0.40	0.01	0.02	0.92
Progesterone venous–arterial difference (nmol/L)	0.47	0.002	0.37	0.02	-0.04	0.82
Net placental release of progesterone (nmol/min)	0.61	< 0.001	0.43	0.005	0.08	0.60

Table 4 shows the steroid release in relation to lactate-adjusted uteroplacental glucose consumption and acetate-equivalent uteroplacental consumption of ketones. The absence of a relation between glycolysis and steroid release was confirmed when uteroplacental glucose consumption was adjusted for lactate production. As seen in Table 4, estradiol and progesterone concentrations in the uterine vein were positively correlated with lactate-adjusted uteroplacental glucose consumption (ρ = 0.36, *p* = 0.05, *n* = 31; ρ = 0.52, *p* = 0.003, *n* = 31, respectively). Similarly, the net placental release of estradiol and progesterone into the maternal circulation was positively associated with lactate-adjusted uteroplacental glucose consumption (ρ = 0. 55, *p* = 0.001, *n* = 31; ρ = 0.60, *p* < 0.001, *n* = 31, respectively). We did not find any significant correlations between steroid hormone release and uteroplacental consumption of acetate-equivalents (ketones) (*p* > 0.05).

**Table 4:** Correlation of steroid hormones with uteroplacental lactate-adjusted glucose consumption and acetate-equivalent ketone consumption

	Lactate-adjusted uteroplacental glucose consumption (µmol/min)			Acetate-equivalent uteroplacental ketone consumption (µmol/min)		
	ρ	*p* value	*n*	ρ	*p* value	*n*
Estradiol (nmol/L), maternal radial artery	− 0.0002	0.999	31	− 0.13	0.49	31
Estradiol (nmol/L), maternal uterine vein	0.36	0.05	31	− 0.06	0.73	31
Estradiol venous − arterial difference (nmol/L)	0.55	0.001	31	0.04	0.84	31
Net placental release of estradiol (nmol/min)	0.55	0.001	31	0.08	0.68	31
Progesterone (nmol/L), maternal radial artery	0.08	0.66	31	− 0.26	0.16	31
Progesterone (nmol/L), maternal uterine vein,	0.52	0.003	31	− 0.10	0.58	31
Progesterone venous–arterial difference (nmol/L)	0.53	0.002	31	0.02	0.90	31
Net placental release of progesterone (nmol/min)	0.60	< 0.001	31	0.004	0.98	31

## Discussion

In this human in vivo study of term pregnancies, we found that uteroplacental uptake and consumption of glucose were associated with the release of steroid hormones into the maternal circulation, whereas no relation to fetal consumption of glucose was found. The net release of steroid hormones into the maternal circulation was neither related to glycolytic glucose consumption nor to placental consumption of acetate (ketones). This conclusion is supported by the finding that the relation between steroid release and glucose consumption persisted after adjustment for lactate production. The latter indicates that energy from acetate is not necessary for steroid production implying that beta-oxidation of fatty acids is not important.

Our findings are compatible with the model that makes non-glycolytic placental use of glucose essential. Based on general biochemical insight, the non-glycolytic pathway is most likely the pentose phosphate pathway that generates NADPH. Our findings also actualize questions dealing with the relation between placental hormone production and maternal glucose metabolism including insulin resistance and diabetes [28]. Interestingly, the net placental release of steroid hormones into the maternal circulation did not show any significant correlations with maternal factors such as maternal age, parity, pre-pregnancy BMI, or gestational weight gain as discussed below. Furthermore, we found no relation between steroid release and placental weight showing that placental weight is not closely related to steroid production.

An inverse correlation between the placental release of steroid hormones into the maternal circulation and glucose concentration in the uterine vein is in accordance with the positive relation between uteroplacental glucose uptake and estradiol release. Previously, we have shown that uteroplacental glucose uptake was not associated with fetal glucose uptake, and this lack of relationship was due to the fact that the placenta’s own handling of glucose affected the net mass of glucose release to the fetus [23, 29]. Considering this together, the findings in the present study are in line with the notion that the placenta utilizes glucose both in glycolytic [14, 15] and in non-glycolytic pathways. The latter includes the pentose phosphate pathway as mentioned. To which extent the hexosamine pathway uses glucose in the placenta is unknown.

**Fig. 2:**
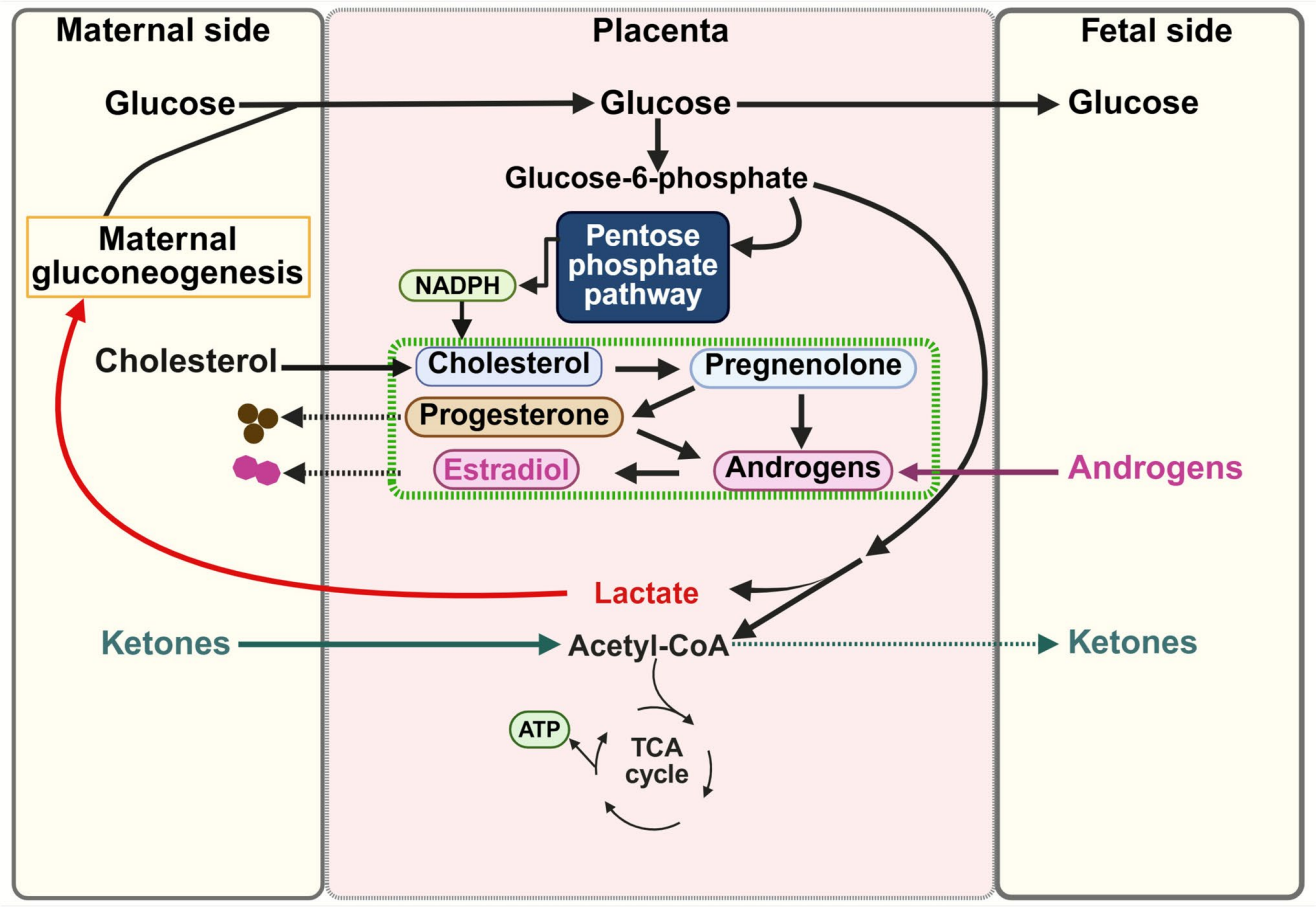
A working model of placental steroidogenesis in relation to placental glucose metabolism. Our model indicates that the placenta utilizes glucose via the pentose phosphate pathway to produce reduced nicotinamide adenine dinucleotide phosphate (NADPH), which is necessary for the steroidogenic pathway. Our data show that the net release of steroid hormones into maternal circulation was not linked to glycolytic glucose consumption or to acetate-equivalent uteroplacental ketone consumption by the uteroplacental tissue. This figure was produced by utilizing the BioRender website (www.biorender.com)

Previously, it has been shown that estradiol is associated with increased NADPH and reduced oxidative stress-induced cell death [30, 31]. In addition, estradiol has also been reported to promote glucose metabolism through the pentose phosphate pathway [32]. These findings imply that NADPH is a source of energy for the placental biosynthesis of steroids as well as regulating the placenta’s own energy homeostasis. Alternatively, the placental access to glucose may stimulate steroidogenesis through the pentose phosphate pathway by producing NADPH (Fig. 2).

Moreover, our data showed a close association between the net placental release of estradiol and progesterone into the maternal circulation in healthy pregnancies. This indicated that the steroidogenic pathways are well-coordinated in the placenta. In addition, the levels of both hormones increase with gestational age [33–35]. A substantial difference in arterial levels of maternal estradiol and progesterone in our study is also in line with previously published work [35]. This difference is likely due to the counterbalancing roles of steroids during pregnancy. For instance, estrogens have a role in cell growth and proliferation, whereas progesterone is crucial for cell proliferation inhibition, differentiation, and maturation [33].

In pregnancies in women with obesity, maternal steroid hormones have been reported to be altered and associated with fetal sex [36]. It has also been shown that maternal insulin resistance increases with gestational age in healthy pregnancies [37]. The role of steroid hormones becomes even more important when it has been shown that normal-weighted women with gestational diabetes mellitus had different steroid hormone profiles as compared to women with normal glucose tolerance [38]. The fact that clinically well-controlled diabetic pregnancies have an increased rate of high birthweights reflects an unrevealed interplay between maternal steroids, placental metabolic and endocrine function, and deviating fetal growth. This interplay may also be operating in non-diabetic pregnancies with deviating fetal growth. There is, therefore, a need for studies of steroid hormone profiles in pregnancies with complications such as deviating fetal growth both with and without gestational diabetes mellitus.

One of the key strengths of this study is the combination of blood samples from the same mother–placenta–fetus triads, Doppler blood flow measurements, maternal steroid hormone data, and NMR spectroscopy-based lactate and ketone data from healthy pregnancies at term. This combination of different methodologies in this study shed light on maternoplacental metabolism and placental endocrine functions as well as steroidogenesis, which allowed us to uncover the association between maternal steroid hormones and placental physiology in healthy pregnancies at term. One of the weaknesses of this study is not having placental enzymatic activity data measuring the pentose phosphate pathway in these pregnancies, although this pathway has previously been shown to be active within the placenta [21]. The lack of correlation between steroid release and placental weight may be attributed to our relatively small sample size and the limited variation in placental weights among healthy pregnancies. This possibility warrants further investigation in a larger cohort. Also, we do not have metabolites (lactate and ketones) data for all the participants, even though their correlations with steroid hormones are robust with existing data.

## Conclusion

There is a strong relationship between uteroplacental glucose consumption and placental release of both estradiol and progesterone into the maternal circulation in the third trimester of pregnancy. The uteroplacental glucose consumption related to steroid production appears to occur mainly via non-glycolytic pathways. Maternal ketones do not seem to be an essential energy source in placental steroid production. This work implies that maternal energy status may influence placental function in terms of steroidogenesis, given that the primary source of glucose for the placenta is derived from the maternal circulation.

## Data Availability

The datasets generated and/or analyzed during the current study are not publicly available due to issues related to informed consent and sensitive data, but are available from the corresponding author on reasonable request.
